# An integrated model of UTAUT2 to understand consumers' 5G technology acceptance using SEM-ANN approach

**DOI:** 10.1038/s41598-022-24532-8

**Published:** 2022-11-21

**Authors:** Sohaib Mustafa, Wen Zhang, Shahzad Anwar, Khalid Jamil, Sehrish Rana

**Affiliations:** 1 College of Economics and Management, Beijing University of Technology, Beijing, 100124 China; 2 MBA Department, Kardan University Kabul, Kabul, Afghanistan; 3 School of Economics and Management, North China Electric Power University, Beijing, 102206 China; 4 Government Islamia Graduate College for Women, Faisalabad, Pakistan

**Keywords:** Human behaviour, Psychology and behaviour

## Abstract

It has been a decade since the first extensive study on the internet's adoption and use was conducted. Circumstances have changed in the last decade internet has become an essential need for every human being. Socio-psychological, economic, and personal factors play a significant role in shaping human behaviour. But their role in shaping consumer behaviour toward 5G is still unexplored. In order to determine the impact of socio-psychological elements on 5G technology adoption intention, the study integrated curiosity, perceived value, functional value, and environmental awareness into UTAUT2 and analyzed how they interact. Instead of relying on linear models, this study employed a dual-stage SEM-ANN approach because customers' decision-making process to adopt new technology is complex. Valid responses from 840 respondents were collected, investigated, and ranked using the deep learning ANN approach. All predictors were found statistically significant except social influence. ANN sensitivity analysis revealed that newly integrated predictors (environmental awareness, curiosity) are surprisingly the most important predictors, followed by facilitating conditions and perceived satisfaction. SEM-ANN hybrid two-step deep learning approach explained 83.6% variance higher than the baseline model (UTAUT2). The study improved UTAUT2 by adding new variables and expanding its canvas to predict user technology adoption. This will show how consumers react to 5G services and help telecoms grow into new markets.

## Introduction

Digital communication technology has substantially evolved since the first analogue devices were introduced in the 1980s. Each generation of digital wireless communication networks has sought to satisfy an increasing human desire (1G, 2G, 3, or 4G). The revolutionary fifth-generation network technology will be available in sixty countries by 2020. The features, as well as performance, far exceed what a rational human would imagine. Elevating the user's overall output unleashes new opportunities and links new industries^1^.

It is anticipated that human civilization would want 5G mobile technology, resulting in an integrated multi-technology and multi-source mobile communication infrastructure capable of meeting future requirements, including development and advancements in networking technologies^2^. For the time being, 5G is deploying faster than 4G. Some researchers say that the mobile market has a short product life cycle; hence customer hesitation may result in low adoption^3^. Future sales projections will be inaccurate if the true level of demand is ignored. The wrong predictions about future demand only hinder the expansion of 5G technology^3^.

Recent studies have focused on the 5G security challenges^4^, technological transition^5^, 5G healthcare solutions^6^, and a practical method for 5G cellular networks to register and authenticate groups of IoT devices remotely^7^. Based on the available literature review, no study has yet investigated the extensive factors behind adopting the *newly launched 5G technology as far as consumers are concerned*. Scholars have studied mobile internet adoption^8^ in 2012 and 3G to 4G diffusion^9^. Technological advancement makes the perception and adoption process more complex than in the past. There is a need to *identify more factors and their influence on consumers' technology adoption, as suggested* by 8 in their famous theory, UTAUT2. Furthermore^10^, suggested adding *environmental awareness to the technology adoption model* to study 5G adoption and use. Dahabiyeh, et al.^11^ Recommended studying the *effect of curiosity on technology adoption* and use. Undoubtedly, intrinsic, economic, psychological, and social factors influence consumers to adopt a new product or technology. These are the causes of either a high or low adoption percentage. Regardless of 5G's benefits over current networks, adopters or consumers of 5G technology are eventually influenced by the factors mentioned in this paragraph. So, there is crucial to empirically investigate the factors influencing a user to adopt a new 5G technology^2^. It will not only help us understand consumer behavior towards 5G adoption, but it also helps telecommunication companies to make a good policy when introducing the 5G technology in new markets.

It has been almost a *decade since a comprehensive technology adoption model was developed and tested on mobile internet adoption*^8^. Mobile internet technology was not that much advanced at that time as right now. Conditions and circumstances have changed. Now it's the era of 5G fast-speed internet technology and smartphones. The use of the internet is very common compared to 2012. Consumers' preferences to use mobile internet are also changed because of the digitally growing world. A decade ago, mobile internet was a luxury, specifically in developing countries, but nowadays, it is one of the necessities of human life.

Many studies have identified several important factors that influence consumers' adoption decisions. "Curiosity" is an essential psychological factor and is significant in human decision-making^2,11,12^. So is "environmental awareness" a significant indicator behind human decision-making^2,10,13,14^. But these *two predictors were never used in any technology adoption model*. The impact of these predictors is still unexplored in the context of technology adoption. Furthermore, factors such as perceived satisfaction^2,15^ and perceived functional value^16,17^ have identified significant predictors behind different technology adoption but have never been investigated in cellular technology adoption. We have integrated these predictors into our proposed model to check the impact of social and psychological factors on 5G technology adoption.

This study exclusively *targets identifying the 5G technology adoption factors and categorizing them based on their importance. The second objective of this study is to integrate the above-mentioned socio-psychological factors in UTAUT2 and check their impact on technology adoption intention and use. Thirdly, is there any change in consumer mobile internet adoption and use science 2012 because of technological advancement and improvement?*

To explore the extensive factors behind the adoption of 5G technology, SEM-ANN has been employed in a state-of-the-art way. This approach is extensively applied to investigate technological adoption and is recommended by several scholars^14,15,18–20^. We implied SEM models with a system of regression equations (OLS, Bootstrap std. errors, robust std. errors) to test the proposed hypothesis in the first step. After the initial test results, we applied an artificial neural network (ANN) approach to predict and rank the factors based on their importance and conclude the final results as recommended^21^.

Results at the first step revealed that perceived performance (PP), perceived functional value (PFV), perceived value (PV), perceived satisfaction (PS), hedonic motivation (HM), curiosity (CUR), environmental awareness (EA), Habit (HAB), and facilitating condition (FC) are significant and positively associated with behavioural intention (BI). Furthermore, BI is significant and positively associated with 5G usage behaviour (UB). In contrast, cost value (CV) is significant but negatively associated with BI. Social influence was found insignificant.

We first applied SEM to test the hypothesis in STATA 15 and ANN using IBM SPSS-25 to rank the significant predictors in the second step. EA and CUR, two factors incorporated for the first time in any technology adoption model, are found 1st and 2nd most influential factors based on sensitivity analysis. FC is the 3rd, and PS is the 4th most significant factor. Sensitivity analysis provided information about the grey areas that can be improved to achieve maximum technology adoption and use.

## Theoretical framework and hypothesis development

To better understand how people use the internet and what influences their decision to adopt 5G technology, we suggested a novel model after integrating new factors in the Unified Theory of Acceptance and Use of Technology (UTAUT2).

### Unified Theory of Acceptance and Use of Technology (UTAUT2)

Numerous technological acceptance models have been established, each with its factors. The work of Venkatesh et al. (2003) aims to review and compare different existing models, establish a theory of adoption, and validate it empirically^2^. UTAUT2 is the advanced version of UTAUT presented in (2003). It is a widely accepted and one of the most famous models for technology adoption. Venkatesh et al. applied their model to adopt mobile internet in 2012, making it the most appropriate model for our study. Venkatesh et al. suggested that other important factors may be discovered in future studies, allowing UTAUT2 to be used in a wider range of consumer technology adoption. More studies need to explore the purpose and behavioural extensions of the UTAUT2 model suggested by Venkatesh and his colleagues 8. The primary measurement models of PE, FC, EE, and SI were validated for video and audio web-enabled teleconferences, databases, and proprietary accounting applications. Many of these innovations can apply to the whole sector, but that doesn't necessarily mean they can have good predictive power because they possess unique characteristics such as speed and convenience.

Furthermore, UTAUT2 asked for internet usage to send SMS, MMS, downloading ringtones and logos, java games, mobile email, etc. It was then an advanced feature to use mobile internet, keeping the speed and quality of internet services. Now mobile internet is used much more than this, such as online shopping, e-payment, navigation, attending conferences and presenting work, social media surfing, etc. In short, we can say the use of the internet, its perceived and actual benefits, and its usages are far more complicated than ever before.

Strong ties to people and a creative spirit often affect how effective and easy a tool is to use. We believe that economic and social factors of individual characteristics influence innovation perceptions and adoption. According to^22^, it is vital to include specific psychological factors affecting decision-making while investigating technology adoption. Researchers recommended incorporating these factors for a successful marketing campaign for new technologies^2^. Thus, to help with current research, we built a model customized for 5G internet technology needs. This model prioritizes discovering the antecedent thoughts and behavioural purposes.

**Figure 1:**
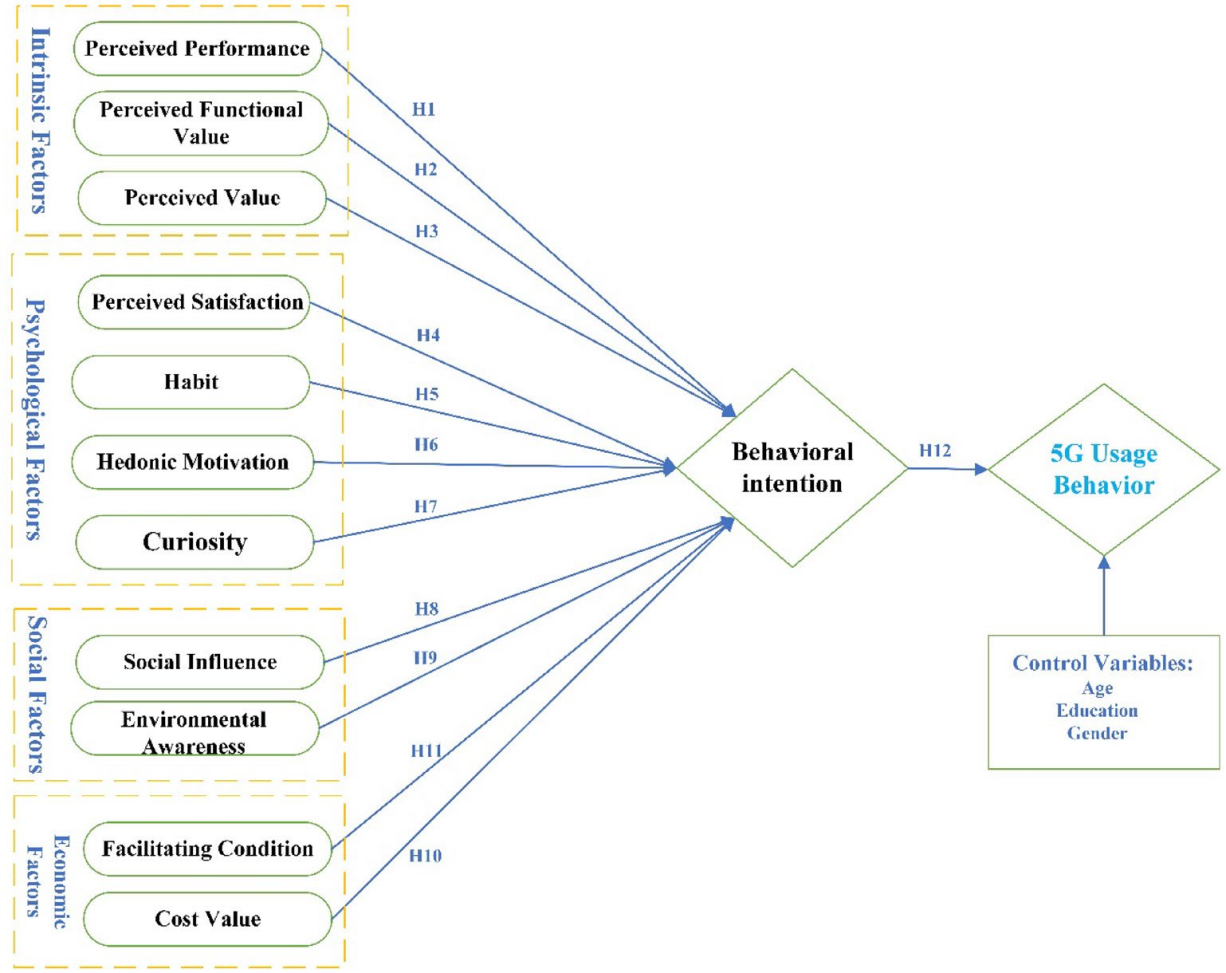
Conceptual Framework.

Model-based on the UTAUT2 to investigate customers' 5G technology usage behaviour with respect to intrinsic, physiological, social, and economic factors is presented in Fig. 1.

#### Newly integrated factors

We have integrated some new factors in UTAUT2 and excluded one factor from the UTAUT2 to better understand consumer technology adoption. Here are the reasons and associations of all the newly identified factors with UTAUT2.

##### Effort expectancy

WE have excluded effort expectancy from the UTAUT2 because of its irrelevancy with a new generation of mobile internet technology and consumers. Questions asked to measure effort expectancy such as. "Learning how to use mobile internet is easy for me. My interaction with the mobile internet is clear and understandable. I find mobile internet easy to use. It is easy for me to become skilful at using mobile internet." These questions were relevant when mobile internet was new for digital immigrants more than a decade ago. But, these questions are quite irrelevant for today's digital native generation because of the advancement and developments. Furthermore, our pilot study identified it as the least concerned variable in our model. Hence, we have decided to drop this.

##### Perceived value

Value-based mobile technology adoption model proposed that perceived benefits of mobile internet and perceived sacrifices are the basic ingredients to make a perceived value of mobile technology that leads to its adoption intentions^23^. We believe that the perceived value of technology plays a vital role in moulding consumers' behavioural intentions to use it permanently. It makes it a more relevant factor in mobile internet adoption; hence, we have integrated it into our proposed model.

##### Perceived functional value

5G mobile internet technology is exponentially known for its better quality and speed. It makes sense that consumers will have an exclusive perception of its quality and speed. Hence, we have decided to identify whether the perceived functional value of 5G plays any role in its adoption decision or not. Compared to effort expectancy, perceived functional value is a much more relevant variable in the current age. According to previous research, people are likelier to adopt new technologies or products with high functional values^17,24,25^.

##### Curiosity

Is well-defined as "a desire for information in the absence of extrinsic reward"^26^. Satisfying one's curiosity is seen as a reward in itself^12^. Existing research found that exposure to liberation from curiosity activates regions in the striatum previously connected with reward-based decision-making^27^.

Two scientific explanations for curiosity have been proposed: drive and incongruity theories^26^. The theory claims that the desire to explore and discover can link to the instinctual drives of thirst and hunger. Curiosity leads to information-seeking, while hunger leads to food consumption. On the other hand, incongruity theory argues that curiosity aroused by incongruity motivates the search for an explanation^11^.

Curiosity is divided into two categories: trait and state. Although trait curiosity represents an individual's proclivity to experience curiosity, state curiosity is situational and contextual and manifests itself only when External conditions have to enable conditions^11^. This analysis focuses exclusively on trait curiosity because it is more consistent with our research goal. Human curiosity has been largely ignored in previous research on user behaviour. According to a recent study, curiosity is a major driving force for behavioural intention^11^.

##### Environmental awareness

Consumers have different opinions about mobile internet technologies. Some consider it a green technology with no side effects, whereas some consumers are worried about the impact of radiation on the ecological system. They believe signals travel in the form of radiation, and the wavelength of these signals can be harmful to human health. Environmentally aware consumers are concerned about green technologies and prefer to use harmless technologies^14^. Recent studies have identified that environmental awareness plays a significant role in purchasing 5G^10^.

### Intrinsic factors and Behavioral intention (BI)

Numerous intrinsic characteristics have been identified as major determinants of behavioural intention. Perceived performance refers to how individuals realize that utilizing an information system will help them achieve performance targets. Venkatesh, et al.^8^ declared perceived performance as the significant forecaster of intention. Perceived performance describes behavioural intentions and functional intent in the literature on mobile applications^28^. Technology adoption is higher when it is easily accessible and simple to use. Perceived performance has significantly predicted mobile phone service usage^2^. Researchers explored perceived performance as an influential factor in mHelth adoption during covid-19 pandemic^18,29^. Technology will probably be accepted if it is beneficial in routine life and reduces human effort while increasing performance and efficiency. We may infer from the information reviewed above that.

#### H1

*Perceived performance is associated with BI using 5G technology.*

Perceived functional value (PFV) refers to the consumers' perception of the quality, performance, and functionality offered by a product or technology^8^. Previous studies have explored that if a technology or product has exceptional functional values, users widely accept it^17,24,30,31^. A recent study explored the association between PFV and a health-consciousness attitude. They argue that PFV is a significant intrinsic factor in shaping consumers' behavioural intention to use technology^2^. The same is found in another study on smartwatch adoption; researchers claim that PFV is a significant factor in explaining the different configurations of consumer smartwatch adoption^32^. So, if a newly introduced technology has the acceptable quality and satisfies the users with its functions and performance, it is expected to adopt instantly. We believe that if the functions of 5G technology will fulfil the perceived functional value of 5G users, they are likely to use 5G technology. These findings dragged us to hypothesize that.

#### H2

*Perceived functional value is associated with BI using 5G technology.*

Perceived value is a person's perception of the benefits certain items or activities can provide compared to their cost^23^. The perceived value used in this study refers to the advantages users expect 5G technology to provide compared to their sacrifices. As long as the user believes a 5G technology has the potential to provide value, even though that value has not yet been realized, that assumption is compatible with the study's concept of perceived value for a 5G technology. In past research, the perceived value of products was referred to as 'hedonic value,' 'utilitarian value,' and 'social value' in smartphones and websites^33^. Some studies have combined these three perceived qualities into a single factor when examining commercial endorsements or live videogame play^34^. Specifically, in 5G purchase intention, perceived value is positively significant with adoption intention^35^. Considering this literature, we hypothesize that.

#### H3

*Perceived value is associated with BI using 5G technology.*

### Psychological factors and BI

The basic fundamental phenomenon of the human psyche is that if a human is satisfied by something, they use it and adopt it permanently. The concept of satisfaction is widely studied in almost every field of social sciences. Motivational theories also emphasize satisfaction for the retention of customers and continuous participation. Satisfaction is categorized as a psychological factor influencing cellular technology adoption^2^. It is also revealed as a significant factor in using online teaching moods during covid-19^15^, m-health adoption^18^, and electronic vehicle adoption^14^. These studies' findings lead us to hypothesize that.

#### H4

*Perceived satisfaction is associated with BI in the use of 5G technology.*

Venkatesh, et al.^8^ well-defined habit as "the extent to which people tend to perform behaviour automatically because of learning." Their behavioural purpose was the most important in predicting technology adoption but could be explained by habit. Wu^35^ describes habit as the result of conditioning due to habitual behaviour. A technological habit is formed when people browse the internet on their smartphones utilizing numerous apps^28^. Habit is also significantly associated with the behavioural intention and health consciousness attitude for technology adoption^2^. We believe that habit positively influences BI in 5G technology. From this, we conclude that:

#### H5

*Habit is associated with BI in the use of 5G technology.*

Hedonic motivation refers to the pleasure or satisfaction derived from new technologies^8^. If people realize there is fun and enjoyment, they will also embrace the technology and be more inclined to use it because they like it. According to many recent studies, hedonic enjoyment of technology increases the desire to embrace and use a service^2,36,37^. Several studies on customer behaviour showed that playfulness and enjoyment are important determinants of customer acceptance of technology^8,18,32^. We also hypothesize that.

#### H6

*Hedonic motivation is associated with BI using 5G technology.*

According to the information gap theory, interest develops as people know how much they know and what they desire to know^38^. This negative state causes an unsettling sensation of incompleteness, eliminated by exploration about filling this void. When looking for facts, one must go to the point of reference. People will work hard if inspired to discover new knowledge and answers. In contrast, they are aware of an old point of reference to keep others working until the point at which they are currently motivated to find answers is reached. Previous studies have shown that people pay for knowledge that satisfies their curiosity^39^ and even takes significant amounts of time to obtain^12^.

The current studies show that curiosity drives actions, and being unaware of those aspects significantly elicits interest^11^. They claim that presenting information about the known dangers piques players' interest and encourages them to look for more information in the sense of the internet and online games. According to recent studies, curiosity is crucial in human behaviour and motivates them to experience e-commerce^19^. These research findings invoke us to present a hypothesis that.

#### H7

*Curiosity is associated with BI in the use of 5G technology.*

### Social factors and BI

Social influence describes the degree to which a person regards the views of others as significant enough to justify adopting the innovative system^8^. Social influence (SI) or conformity also refers to the strain other people apply to a person's beliefs^32^. Indeed, technology acceptance is highly dependent on an individual's thinking and social impact^2^. It is based on the hypothesis that users' behaviour derives from a prior ability. They perceive how others perceive their use of technology^19^. Adoption decisions are socially influenced by a person's or group's available position. The social effect positively impacts technology usage^2^. It has also found that it has no influence when it comes to e-commerce^20^. Thus, we argue that.

#### H8

*Social influence is associated with BI in using 5G technology.*

Care for the environment includes environmental concerns, strategies, and ideas for tackling environmental issues^13,40^. Consequently, environmental consciousness is essential in individuals' transformation from their current actions to more sustainable behaviour^13^. Consumers more conscious of the environment are likely to pay more than those with less knowledge^19^. The study created an expanded TPB model to find that environmental factors significantly predict customer intent 19. It also affirmed attitude and actions. Without positive ecological or health benefits, people are unlikely to pay attention to environmental and health considerations^14^. In 5G technology, environmental awareness is a significant antecedent behind its purchase 10. Previous investigation has led us to believe that.

#### H9

*Environmental awareness influences consumers' BI using 5G technology.*

### Economic factors and BI

Cost value entails reasonable prices and the best possible value for money^32^. The 'Cost value' indicates which application a consumer believes offers a better value for money when compared to the whole cost^8^. According to a rational choice principle, price value balances the overall expense and the future gains in operation^41^. Researchers have found that cost value is a determinant factor in purchasing eco-friendly products^19^ and smartwatches^32^. In the context of mHealth adoption during the covid-19 pandemic, the cost value is the significant predictor^18^. These studies influence us to hypothesize that.

#### H10

*Cost value is negatively associated with BI using 5G technology.*

Venkatesh, et al.^8^ Describe facilitating conditions as "the degree to which an individual believes that an organizational and technical infrastructure exists to support the use of the system.". Facilitating conditions affect the acceptance of e-commerce in developing countries^20^ and the adoption and use of eco-friendly products in developing countries^19^. It is also found to be positively associated with the adoption intention of technology and the health-conscious attitude of consumers toward technology adoption^2^ Healthcare technology adoption facilitating conditions are revealed as a significant indicator^18^. These studies lead us to hypothesize.

#### H11

*Facilitating conditions are associated with BI using 5G technology.*

### Behavioural Intention (BI) and Usage Behavior (UB)

This study's model compares the adoption of 5G technology to earlier models and their expected construct. But understanding user purpose and behaviour is critical to technology adoption. The most important factor is the intention behind real usage^2^. A core premise of adoption is that a person's 'intention' to apply new technology is projected by its 'actual usage'^2,8,42^. Several scholars used "intention to use" and "actual use" as explained variables in technological adoption studies^8,18^. The researchers have used real actions and behavioural intent interchangeably as explained variables. According to past research, individuals who believe they can utilize new technology are inclined to use it^42^. Venkatesh, et al.^8^ found that "behavioural intention" and "actual use" are similarly important for technology use. These two primary factors are used to gauge the acceptability of technology in literature^18^. According to the literature, the usage study of technology is almost impossible to separate from intentions. Technology adoption is intimately linked to an individual's response to utilizing it. Thus, consumption impacts "usage behaviour"^8,43^. According to the existing literature, behavioural intention (BI) predicts this study's 5G technology usage behaviour.

#### H12

*BI is associated with the 5G usage behaviour.*

### Control variables

Demographics is an essential factor in consumers' choice, either positive or negative, of adopting a new product or technology^44^. Most research on the impact of demographics has concentrated on innovation acceptance. It is essential and crucial to study demographics to ensure 5G adoption. People are increasingly reluctant to use online or mobile banking in older age^45^. Several previous studies have considered age and gender as antecedents^43^, moderator^8,24^, and control variables 30 in technology adoption studies. Education level is also considered a control variable to check technology adoption^11^. Users' propensity to adopt internet services is a feature of their level of education^44^. Age, education, and gender have significant influences in these studies. As a result, we expect age and gender to negatively impact 5G technology usage.

## Methodology

### Research context

According to a Huawei Technologies Co. official, China's 5G user penetration rate has surpassed the 20% mark, indicating rapid development of 5G^46^. In a survey conducted in December 2019 by Daniel Slotta and published in 2020, Chinese people are generally well-informed about and open to new technology^47^. They also anticipated that in 2022, a 289.3% growth rate is expected in 5G users from China. The population of china also makes it the biggest market for any technology. Hence we have decided to test our model in china. To ensure the validity of our findings, we interviewed Chinese residents and foreigners working, studying, or researching in China. We distributed our questionnaire in all major cities of China to collect the best data and maintain the uniformity and generalizability of results.

### Instrument development & data collection

We have adopted a valid construct from previous studies. Detailed construct items and references for each measurement item are provided in the supplementary material. We modified the construct items slightly to make them more effective for our study objective. We present the instrument to three academic experts and incorporate all the expert opinions. We ran a pilot study to ensure that respondents' consumption time and comments did not adversely influence the survey's quality as suggested^48^. Pilot research included^28^ master's and Ph.D. students. During the pilot study, every feedback we received was again consulted with the academic experts and incorporated the necessary suggested material. We consulted two language experts to translate our instrument into Chinese to facilitate the target population at the next stage. Language experts translate the English version of the instrument into Chinese. Chinese translated version of the instrument again consulted with academic experts to ensure that constructed items have the same meaning in English and Chinese. Finally,^20^ Chinese master's students were chosen for the final instrument testing. They gave favourable comments, and the second phase pilot research findings were encouraging—the pilot study contributors were not involved in the total sample.

We used an online survey to avoid human mistakes. We approached different expat online communities to collect answers from foreigners living in china. Furthermore, according to shah et al.^10^, students possess a high level of knowledge and awareness regarding environmental issues, so we have decided to get a response from students. This study classifies individuals using non-probability sampling. It is a good approach to offering an overall perspective of phenomena^49^. Respondents were prompted to enter their mobile phone numbers to weed out those who had taken the poll more than once. The professional online questionnaire star Chinese was used to conduct a formal online survey (www.wjx.cn).

**Figure 2:**
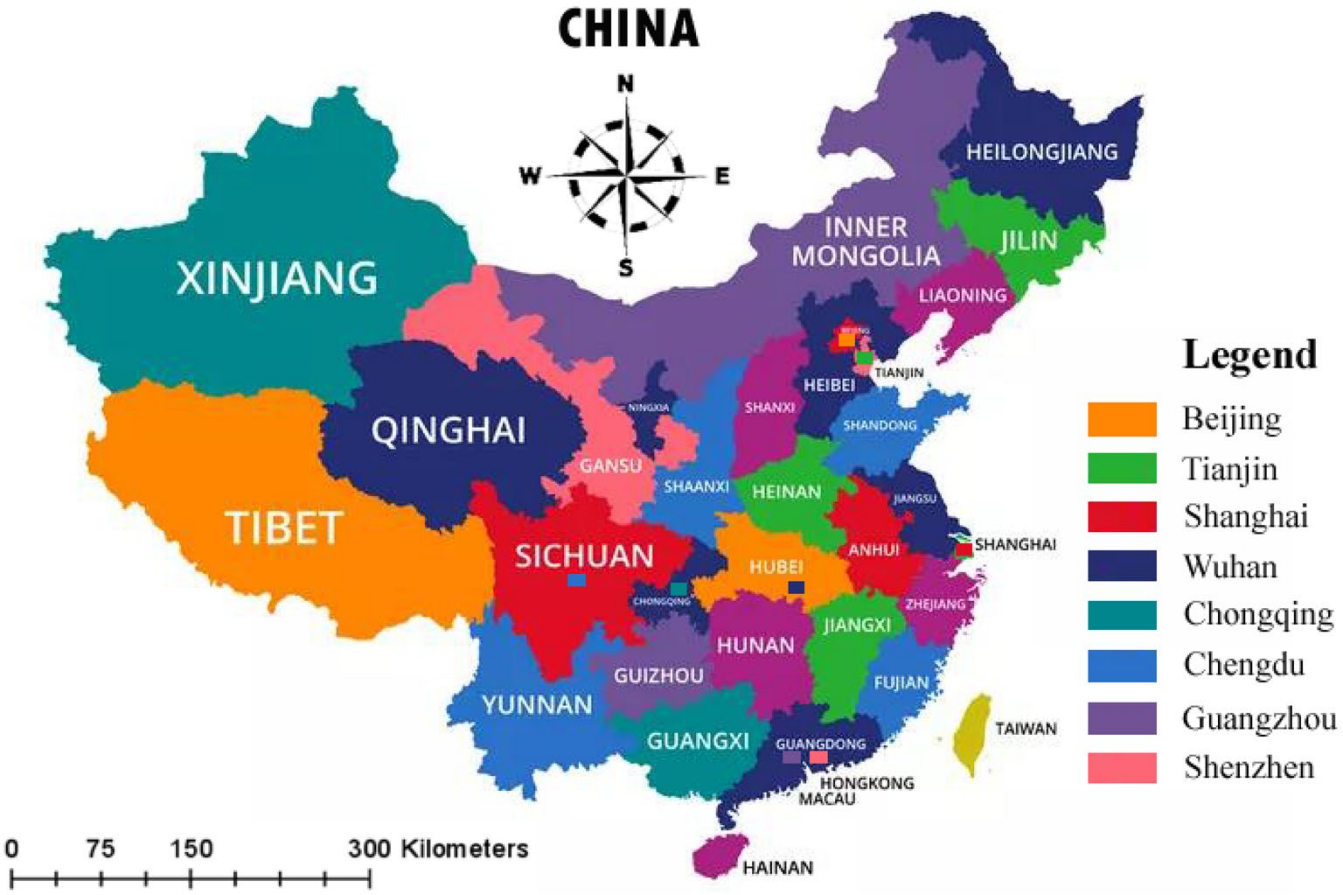
Study area: Authors have downloaded a free version of the Chinese map available at https://graph.baidu.com/ and used Adobe PhotoShop CS5 to incorporate legend. The legend shows the name of Chinese cities where the survey was conducted to collect the dataset for analysis.

We have selected five points in each key city (Fig. 2) of china to reach the target population (Shopping malls where 5 g enabled cell phones are sold and offices of telecommunication companies). Fourteen hundred questionnaires via QR code were distributed between the first three weeks of September 2021. Respondents were informed about the purpose of the study. It took four weeks to collect a valid response of 840. All the variables were measured on a 7-point Likert scale where 1 as "strongly disagree" and 7 as "strongly agree." 7-point Likert scales are more precise, simpler to apply, and more accurate. As a result of these advantages, seven-point survey questions tend to be better than their higher-order alternatives^50^. The sample size of 840 is much more than ten times that of the arrows pointing to an endogenous variable and is thus suitable for statistical analysis^44,51^.

### Respondents' demographic profile

The demographics of 840 respondents, such as gender, age, education, occupation, Residential Status, Technology User, and Tenure Using 5G, are presented in Table 1.

**Table 1:** Demographic Information.

Characteristics	Range	Frequency
Gender	Male	449
	Female	391
Age	18–25Year	205
	26–35Year	275
	36–45Year	279
	> 45Year	81
Education	High school or less	137
	Bachelor	325
	Master	352
	Doctorate	26
Occupation	Student	217
	Govt. employee	174
	Private company employee	266
	Businessman/women/other	183
Residential status	Chinese citizen	505
	Expatriate	335
Technology user	4G	297
	5G	543
Tenure using 5G	< 3 Month	115
	3–6 Months	222
	> 6 Months	206
	None	297
	Total	840

### Common method bias (CMB)

Since the data for predictor and outcome constructs were obtained from the same respondent using a similar instrument, common method bias is possible. Procedures and statistics were used to deal with CMB. Respondents were informed that their identities would be obscured and that there were no definite right or incorrect answers, so they may react candidly^52^. In addition, the different scale was used in the instrument. E.g., gender, Residential status, and technology use were captured using a nominal scale, whereas age, tenure using technology, education, and occupation were measured using a categorical scale. All exploratory and explained measures were captured using 7-point Likert interval scales. Finally, Harman's single-factor analysis was performed, and the analysis revealed that a single factor only explains 20.11, 43.73% of the total variance. Since it is less than 50% of the threshold limit, no problem exists with the CMB^51^. Furthermore, the correlation coefficient (Table 2) indicates less than 0.90 means there is no CMB issue^51^.

**Table 2:** Discriminant Validity.

	Mean	Std.dev	BI	CUR	CV	EA	FC	HAB	HM	PFV	PP	PS	PV	SI	UB
BI	4.94	1.435	*0.901*												
CUR	5.209	1.33	0.632	*0.882*											
CV	4.921	1.351	0.565	0.800	*0.870*										
EA	4.596	1.392	0.697	0.473	0.421	*0.765*									
FC	4.875	1.326	0.819	0.645	0.645	0.623	*0.846*								
HAB	5.298	1.207	0.588	0.749	0.656	0.416	0.632	*0.853*							
HM	5.204	1.351	0.686	0.531	0.554	0.503	0.739	0.637	*0.900*						
PFV	5.201	1.431	0.627	0.476	0.408	0.444	0.568	0.519	0.496	*0.930*					
PP	4.661	1.457	0.769	0.562	0.570	0.614	0.778	0.548	0.584	0.556	*0.879*				
PS	4.526	1.332	0.720	0.496	0.499	0.538	0.759	0.532	0.511	0.523	0.762	*0.776*			
PV	4.349	0.978	0.252	0.178	0.177	-0.010	0.214	0.143	0.178	0.291	0.203	0.167	*0.852*		
SI	5.053	1.203	0.566	0.737	0.640	0.397	0.603	0.786	0.565	0.440	0.548	0.544	0.172	*0.803*	
UB	4.953	1.452	0.885	0.623	0.577	0.722	0.807	0.565	0.637	0.587	0.710	0.689	0.271	0.558	*0.906*

PP = Perceived Performance; PFV = Perceived Functional Value; PV = Perceived Value; PS = Perceived Satisfaction; HAB = Habit.

Hedonic Motivation; CUR = Curiosity; SI = Social Influence; EA = Environmental Awareness; FC = Facilitating Condition;

CV = Cost Value; BI = Behavioral Intention; UB = Usage Behavior. Significant values are in italics.

### Multivariate assumptions

According to^53^, the multivariate assumptions of linearity, normality, homoscedasticity, and multicollinearity must be examined before doing any multivariate tests. The data normality distribution has been ascertained using the One-Sample Kolmogorov–Smirnov (K-S) test, but the findings show that the data is not normally distributed^54^ (see supplementary material). Linear and nonlinear interactions between dependent and independent constructs are presented in the supplementary material. This study examined the variance inflation factor (VIF) scores to determine collinearity issues with the model. According to^51^, if the VIF values are less than 5, this implies that there are no collinearity problems in the gathered data. According to the findings of this analysis, the values of VIF for all variables are less than 5. It confirms no collinearity in the current study data and reinforces the model's robustness. And then, the scatter plot of the regression normalized predicted and residual values shows that this prediction is met^20^.

### SEM-ANN

There are well-known fit measures for regression systems and various other metrics to understand better how each estimator performs. These metrics include the residuals, outliers, and leverage of individual data items on estimators. In contrast to the unscaled path coefficients of the PLS-PA and AMOS-LISREL techniques^55^, recommends scaled regression estimators. Hence, we have adopted SEM to test the proposed hypothesis.

According to^56^, if the data has a non-normal distribution, variance-based SEM is an excellent approach compared to factor-based SEM or PLS-SEM. Since the model contains nonlinear relations, it is preferable to conduct a two-step analysis, as suggested by Mustafa and Zhang 56. The ANN research is carried out after the SEM (in STATA 15) because approaches, including OLS, composite-based SEM, and factor-based SEM, cannot handle nonlinear relationships among the constructs^54^.

As proposed^54^, ANN is a simulation technique replicating human neural network models and gaining information via its learning method. Furthermore^54^, stated that ANN could learn to improve its efficiency, given its learning capacity. The ability to learn separates ANN from other multivariate analyses, such as SEM, which makes it unique in its prediction and consistency in outcome^20^. Besides that, including traditional multivariate analyses such as SEM, ANN's "black-box" procedure prevents it from determining the significance of causal interactions, making it unsuitable for hypothesis testing^53^. Researchers have recommended that it is advantageous to mix both SEM and ANN studies to benefit from the advantages of both distinctive analysis methods^14,20^. Many researchers have recognized that these analyses may balance and produce a more comprehensive data analysis^53^. According to standard procedures for performing a dual analysis, SEM in STATA 15 has been used first to determine the substantial exogenous constructs used as input neurons in the ANN investigation to understand the non-linearity across the predictors observed constructs^57^ completely. The ANN research was carried out in this study employing IBM SPSS-25.

## Analysis and Results

### Measurement model assessment

The current research model is based on 46 items from the 13 variables, as demonstrated in Table 3. The instrument's Reliability and Validity have been evaluated using the measurement model^51^. It is suggested that measurement models be evaluated based on their reliability as an indicator and concept and their convergent and discriminant validity. The dependability of the instrument was determined using Cronbach's Alpha (α) and indication loading. Convergent validity measures a construct's ability to evaluate the research variables appropriately. AVE is the latent construct's overall variation of the indicators, while CR denotes the variables' consistency. Loadings are higher than the 0.6 thresholds considered to establish the indicator's reliability^51^. For this study, all the items contain factor loading higher than 0.6 except SI4. The average variance extracted (AVE) values are more than 0.50 for all constructs (Table 3), representing the convergent validity of the construct. Additionally, α and CR were greater than 0.7, demonstrating that all latent variables' internal consistency was established^51^. Furthermore, factor analysis results are presented in the supplementary material.

**Table 3:** Measurement Model Results.

Variables	Items	Loadings	T statistics	VIF	α	CR	AVE
Perceived functional value	PFV1	0.922***	110.04	2.136	0.843	0.927	0.864
	PFV2	0.937***	158.24	2.136			
Perceived performance	PP1	0.875***	70.169	2.102	0.853	0.911	0.773
	PP2	0.874***	66.811	2.073			
	PP3	0.889***	99.826	2.141			
Perceived value	PV1	0.874***	62.430	2.504	0.874	0.913	0.725
	PV2	0.880***	55.939	2.688			
	PV3	0.851***	34.886	2.451			
	PV4	0.799***	29.843	1.892			
Perceived satisfaction	PS1	0.786***	38.248	2.084	0.783	0.858	0.602
	PS2	0.730***	27.640	1.918			
	PS3	0.760***	31.606	1.507			
	PS4	0.824***	66.488	1.675			
Habit	HAB1	0.894***	73.058	3.743	0.871	0.914	0.728
	HAB2	0.916***	109.99	3.623			
	HAB3	0.890***	85.588	3.603			
	HAB4	0.694***	27.329	1.276			
Hedonic motivation	HM1	0.835***	42.882	2.022	0.882	0.927	0.809
	HM2	0.944***	210.92	3.680			
	HM3	0.916***	90.105	3.006			
Curiosity	CUR1	0.861***	69.558	2.611	0.905	0.933	0.778
	CUR2	0.915***	135.14	3.484			
	CUR3	0.884***	69.513	3.785			
	CUR4	0.867***	54.396	3.522			
Social influence	SI1	0.844***	65.581	2.490	0.808	0.877	0.644
	SI2	0.866***	67.729	2.512			
	SI3	0.873***	89.948	2.115			
	SI4	0.595***	18.008	1.294			
Environmental awareness	EA1	0.739***	33.253	1.449	0.765	0.85	0.586
	EA2	0.741***	32.328	1.441			
	EA3	0.787***	47.074	1.543			
	EA4	0.791***	53.627	1.491			
Facilitating condition	FC1	0.859***	66.946	2.640	0.899	0.926	0.716
	FC2	0.879***	80.175	2.942			
	FC3	0.863***	64.336	2.773			
	FC4	0.895***	114.63	3.148			
	FC5	0.722***	34.412	1.621			
Cost value	CV1	0.830***	45.321	1.769	0.839	0.903	0.756
	CV2	0.878***	67.027	2.163			
	CV3	0.899***	86.676	2.132			
Behavioral intention	BI1	0.899***	89.424	2.285	0.884	0.928	0.811
	BI2	0.908***	110.53	2.759			
	BI3	0.895***	82.953	2.571			
Usage behavior	UB1	0.889***	76.608	2.489	0.891	0.932	0.821
	UB2	0.905***	83.030	2.651			
	UB3	0.924***	167.29	2.758			

Cα > 0.7; CR > 0.7; AVE > 0.5; VIF < 5; ***Significant at *p* < 0.001.

Discriminant validity is used to determine whether an indicator's correlation score with its measure is higher than the correlation score with other measures. Discriminant validity was ascertained in this analysis using Fornell and Larcker's criteria, which specified that the square root of each construct's AVE must be greater than its highest correlation to every other construct^51^. The diagonal line values are derived by taking the square root of each AVE value. All diagonal AVE values are greater than the other correlation values in the construct correlation matrix, as seen in Table 2.

This study measured the Effect size (*F*^*2*^), predictive power of the proposed model leveraging the identified variance (*R*^*2*^) values, and predictive relevance (*Q*^*2*^) before conducting the hypotheses tests. According to^53^, an effect size of a latent construct varying from 0.02, 0.15, and 0.35 is deemed small, medium, and high, respectively. For this study, Perceived Performance, Perceived Functional Value, Perceived Value, Perceived Satisfaction, Hedonic Motivation, Curiosity, Environmental Awareness, Facilitating conditions Behavioral Intention have medium to high effects. In contrast, the effect size for Habit, Social Influence, and Cost Value are less than the small threshold. Both endogenous constructs have *R*^*2*^ values greater than 0.5, which indicates a robust model^51^. The two latent variables' Q^2^ values (cross-validated redundancy) are substantially greater than zero, demonstrating the model's significance^51^ (Table 4).

**Table 4:** Effect size, Predictive relevance, and coefficient of determination.

	F^2^		R^2^	Q^2^
	BI	UB		
BI		0.627	0.791	0.640
CUR	0.045			
CV	0.015			
EA	0.138			
FC	0.050			
HAB	0.004			
HM	0.051			
PFV	0.036			
PP	0.033			
PS	0.031			
PV	0.028			
SI	0.001			
UB			0.751	0.634

### Path analysis results

Table 5 presents the path analysis results. We have stepwise entered the variables in the models. In model 1, OLS is applied to exploratory variables. In model 2, control variables are also added to the OLS model. As the data is not normally distributed, we have applied robust standard errors to cater to the issue in model 3.

**Table 5:** Path Results.

	OLS(m1)		OLS(m2)		Robust(m3)		Bootstrap(m4)	
BI	Coef	T values	Coef	T values	Coef	Z values	Coef	Z values
PP	0.164***	5.69	0.163***	4.72	0.166***	4.77	0.166***	4.70
PFV	0.123***	5.76	0.123***	5.04	0.123***	5.08	0.123***	5.03
PV	0.11***	4.35	0.11***	4.19	0.075***	4.23	0.075***	4.16
PS	0.134***	4.52	0.134***	3.46	0.124***	3.53	0.124***	3.46
HAB	0.071**	1.98	0.074*	1.92	0.059*	1.86	0.059*	1.82
CUR	0.204***	5.69	0.207***	5.53	0.189***	5.53	0.189***	5.57
EA	0.231***	10.18	0.232***	9.03	0.223***	9.03	0.224***	9.03
HM	0.171***	6.22	0.171***	5.85	0.160***	5.85	0.161***	5.79
CV	−0.105***	−3.61	−0.106***	−4.02	−0.096***	−4.01	−0.098***	−4.00
FC	0.252***	6.56	0.254***	5.40	0.234***	5.40	0.234***	5.36
SI	−0.007 ^NS^	−0.20	−0.006 ^NS^	−0.18	−0.006 ^NS^	−0.18	−0.006 ^NS^	−0.18
Gender			0.046 ^NS^	1.02	0.046 ^NS^	1.02	0.046 ^NS^	1.00
Age			−0.004 ^NS^	−0.18	−0.004 ^NS^	−0.18	−0.004 ^NS^	−0.18
Education			0.012 ^NS^	0.43	0.012 ^NS^	0.43	0.012 ^NS^	0.43
R^2^	0.789	0.790	0.790	0.790				
Adj R^2^	0.787	0.786						
Prob > F	0.000	0.000	0.000					
Prob > chi^2^							0.000	

UB	M5		M6		M7		M8	
BI			0.875***	55.17	0.864***	72.43	0.864***	72.08
Gender	−0.183*	−1.83	−0.104**	−2.06	−0.035**	−2.06	−0.036**	−2.03
Age	−0.018 ^NS^	−0.34	−0.005 ^NS^	−0.21	−0.005 ^NS^	−0.21	−0.006 ^NS^	−0.21
Education	0.095 ^NS^	1.48	0.169 ^NS^	0.56	0.169 ^NS^	0.56	0.017 ^NS^	0.57
R^2^	0.006	0.751	0.751	0.751				
Adj R^2^	0.003	0.750						
Prob > F	0.000	0.000	0.000					
Prob > chi^2^							0.000	
Obs	840	840	840	840				

1. *** p < .01, ** p < .05, * p < *.1; NS* = *Not supported.*

2. PP = Perceived Performance; PFV = Perceived Functional Value; PV = Perceived Value; PS = Perceived Satisfaction; HAB = Habit. Hedonic Motivation; CUR = Curiosity; SI = Social Influence; EA = Environmental Awareness; FC = Facilitating Condition; CV = Cost Value; BI = Behavioral Intention; UB = Usage Behavior.

Furthermore, we have applied the Bootstrap model with 5000 resampling to check the consistency of results in model 4^58^. In the second stage, we changed the explained variable from behavioural intention to use behaviour and tested the second part of our model (M5-M8). Our results are highly consistent throughout the different analysis techniques, implying the validity and consistency of our model.

Findings show that all of the three dimensions, including Perceived performance (β = 0.166, T = 4.77, *p* < 0.001), Perceived Functional Value (β = 0.123, T = 5.08, *p* < 0.001), Perceived Value (β = 0.075, T = 4.23, *p* < 0.001), are significantly associated with behavioral intention, which supports H1 H2 and H3 of our Study. Furthermore, H4 to H7 presented the Psychological factor's impact on behavioral intention. The finding of the analysis revealed that Psychological factors, including Perceived Satisfaction (β = 0.124, T = 3.53, *p* < 0.001), Hedonic Motivation (β = 0.160, T = 5.86, *p* < 0.001), Curiosity (β = 0.189, T = 5.53, *p* < 0.001), and habit (β = 0.059, T = 1.86, *p* < 0.1) are significantly related to the behavioural intention which supports H4 to H7. For social factors, Environmental awareness (β = 0.223, T = 9.03, *p* < 0.001) significantly affects BI, but Social influence does not, which means H9 is supported but H8 is not.

The study also tests the economic factors' impact on behavioral intention. For H10 and H11, findings revealed a significant effect of facilitating condition (β = 0.234, T = 5.40, *p* < 0.001) and cost value (β = −0.096, T = −4.01, *p* < 0.001) on behavioural intention. Even though the impact of cost value on behavioural intention was negatively significant, it is consistent with the previous study, and results for H10 and H11 support the study^59^. In the last hypothesis of the study, we tested the impact of behavioural intention on 5G use behaviour and found a significant relationship between the latent constructs (β = 0.864, T = 72.43, *p* < 0.001). Hence H12 was also supported.

Lastly, findings in the second stage (M6 to M8) exposed that gender as a control variable has a statistically significant impact on User behaviour, whereas age and education have no impact.

We have applied Tobit regression for further robustness and consistency check. The results presented in Table 6 with the bootstrap (M10) and robust (M11) model are highly consistent with the results presented in our main analysis (Table 5 robust standard errors). Identical results presented in Table 6 suggest that our results are consistent and robust with the variation of the analysis technique.

**Table 6:** Tobit regression.

	Robust(m9)		Bootstrap(m10)		Robust(m11)	
BI	Coef	t-values	Coef	t-values	Coef	t-values
PP	0.18***	4.94	0.178***	4.90	0.178***	4.90
PFV	0.132***	5.21	0.132***	5.11	0.132***	5.22
PV	0.106***	3.80	0.106***	3.75	0.106***	3.81
PS	0.126***	3.06	0.127***	3.06	0.127***	3.08
HAB	0.08**	1.97	0.084**	2.07	0.084**	2.07
CUR	0.206***	5.33	0.209***	5.33	0.209***	5.41
EA	0.242***	8.72	0.242***	8.75	0.242***	8.75
HM	0.17***	5.31	0.169***	5.28	0.169***	5.30
CV	−0.102***	−3.66	−0.103***	−3.66	−0.103***	−3.71
FC	0.275***	5.45	0.278***	5.43	0.278***	5.52
SI	0.002 ^NS^	0.05	0.003 ^NS^	0.07	0.003 ^NS^	0.07
Gender			0.057 ^NS^	1.17	0.057 ^NS^	1.18
Age			−0.004 ^NS^	−0.17	−0.004 ^NS^	−0.17
Education			0.013 ^NS^	0.43	0.013 ^NS^	0.43
Pseudo R^2^	0.421	0.422	0.422			
Prob > chi^2^			0.000			
Prob > F	0.000			0.000		

UB	M12		M13		M14	
BI			0.926***	47.60	0.927***	47.94
Gender	−0.173 ^NS^	−1.56	−0.092*	−1.68	−0.092*	−1.69
Age	−0.016 ^NS^	−0.28	−0.005 ^NS^	−0.16	−0.004 ^NS^	−0.16
Education	0.105 ^NS^	1.53	0.033 ^NS^	0.55	0.018 ^NS^	0.56
Pseudo R^2^	0.0016	0.371	0.422			
Prob > F	0.141			0.000		
Prob > chi^2^			0.000			
Obs	840	840	840			

*** p < .01, ** p < .05, * p < *.1; NS* = *Not supported.*

### Goodness of fit

According to the values explained by 59 for the best model fit for path analysis, Table 7 indicates our model's goodness of fit indices. Values show that our model best fits data and results are reliable.

**Table 7:** The goodness of fit Statistics.

	Population error		Baseline comparison		Size of residuals
	RMSEA	pclose	CFI	TLI	SRMR
BI	0.000	1.000	1.000	1.000	0.000
UB	0.017	0.859	0.907	0.910	0.021
Recommended value	Values < 0.05		Values = or > 0.90	Values = or > 0.90	Values < 0.05 up to 0.10

### ANN analysis

We used an ANN with a multi-layer perceptron, a vastly sequential computing processor composed of specific processing units with a neural tendency to preserve and render accessible experimental information^60^. Neurons or nodes are the simplest processing units in an ANN. According to^18^, the obtained information from the context is contained in the weights of the integrated neurons, commonly known as synaptic weights. As indicated by^53^, During the learning phase, data in the input neuron nodes are routed to the outgoing neuron node by the remote neuron nodes using a nonlinear activation feature. Furthermore^53^, specified that synaptic weights would be regulated to achieve the target outcome during the learning phase. Hidden layer neuron nodes train to address the input neuron nodes in an easy-to-predict manner to determine the output neuron node. For example, a shallow ANN design permits shallow learning, whereas a deep one provides deeper learning.

We have developed a two-layer deep-ANN architecture for each output neuron node to enable deeper learning as advised^15,19,20^. The ANN model has been constructed for Behavioral intention in this research, as illustrated in Fig. 4. The sigmoid activation method was used, and the number of hidden neuron nodes was enabled to generate automatically, as proposed^53^. Following recommendation^21^, a tenfold cross-validation protocol was utilized to minimize over-fitting, with 90% of the data used for training and the remaining 10% for testing.

**Figure 3:**
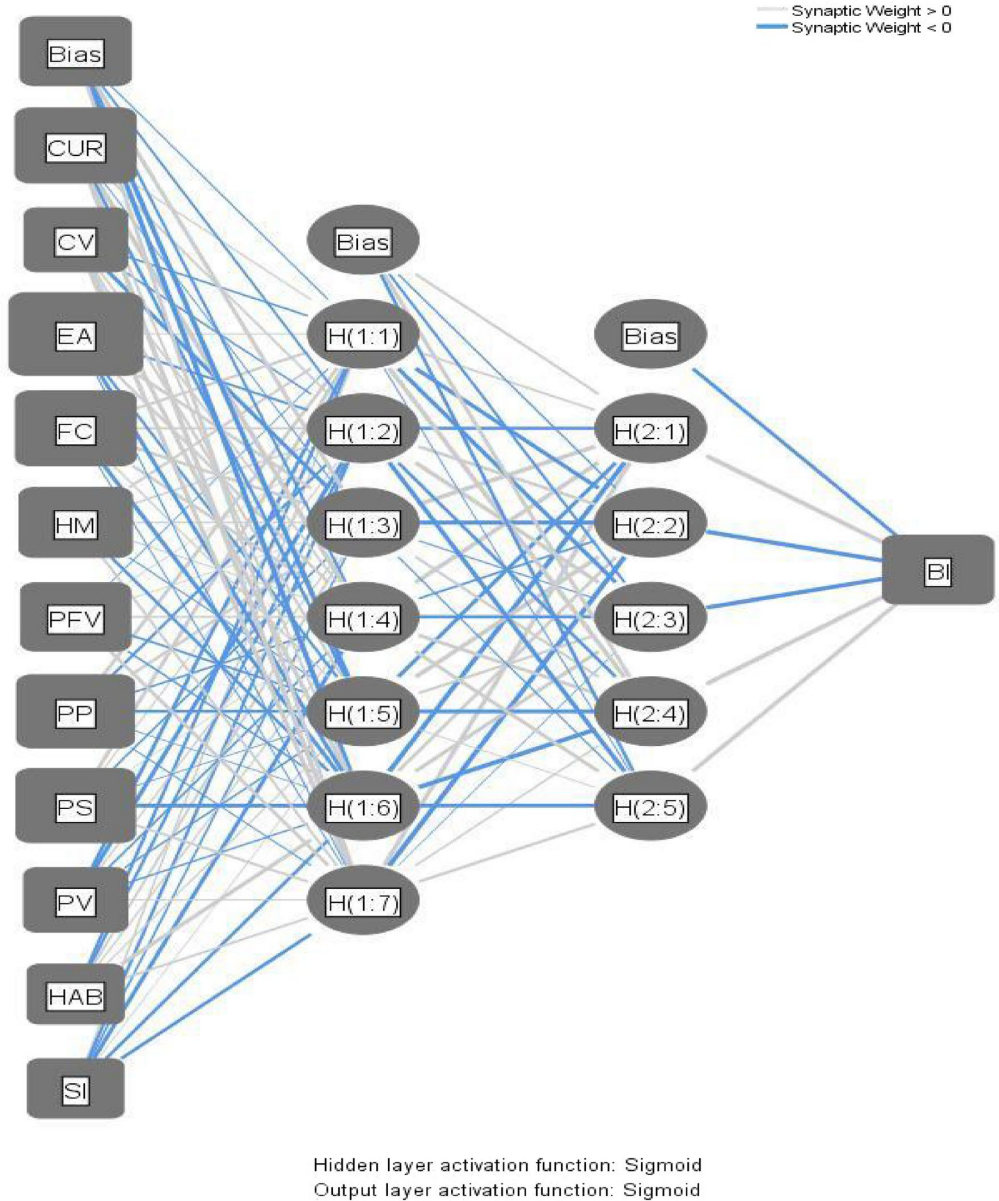
Normalized Importance %.

Table 8 presents the predictive performance of the ANN model. The ANN model has a strong predictive efficiency since the root mean square error (RMSE) values are marginal. Furthermore, we measured a goodness-of-fit coefficient comparable to the R^2^ in the regression models to test the efficiency of the ANN models based on a methodology proposed^53^. The results demonstrate that the R^2^ of the ANN model indicates behavioural intention with a precision of 83.6%. The R^2^ values in the ANN model are substantially higher than those in the SEM models, indicating that the predictor variables are better demonstrated in the ANN analysis compared to the SEM models. We conclude that this result primarily accounts for the two-deep learning architectures and the potential of ANN to capture nonlinear relationships.

**Table 8:** RMSE values.

Training			Testing			Total sample
N	SSE	RMSE	N	SSE	RMSE	
747	4.048	0.074	93	0.565	0.078	840
761	3.771	0.070	79	0.494	0.079	840
750	4.011	0.073	90	0.376	0.065	840
771	3.865	0.071	69	0.295	0.065	840
762	4.690	0.078	78	0.336	0.066	840
745	3.594	0.069	95	0.464	0.070	840
761	3.656	0.069	79	0.426	0.073	840
746	3.778	0.071	94	0.466	0.070	840
761	3.839	0.071	79	0.466	0.077	840
753	3.464	0.068	87	0.407	0.068	840
Mean	3.872	0.072	Mean	0.4295	0.0711	
Std Dev	0.338	0.002951	Std Dev	0.0792	0.0053	

1. *R*^*2*^ = 1-RMSE/S^2^, where S^2^ is the variance of the test data's desired output. 2. N = number of samples, RMSE = root mean square of errors. 3. Perceived Performance; Perceived Functional Value; Perceived Value; Perceived Satisfaction; Hedonic Motivation; Curiosity; Social Influence; Environmental Awareness; Facilitating Condition, Cost Value served as the input neurons. 4. Behavioural intention served as the output neuron.

The guideline of^53^ determines each input neuron's predictive capabilities (Table 9). Calculate the normalized value of these neurons by dividing their relative importance by the highest possible significance and expressing it as a percentage. The findings illustrate that EA is the most important predictor with normalized importance of 100%, followed by CUR (94.8%), FC (89.5%), PS (88.4%), HM (63.3%), PV (42.5%), PP (40.7%), PFV (38.6%), HAB (31.4), CV (30.6%), and SI (27.6%) (Fig. 3).

**Table 9:** Sensitivity analysis.

Neural network	CUR	CV	EA	FC	HM	PFV	PP	PS	PV	HAB	***SI***
NN-1	0.125	0.056	0.173	0.125	0.093	0.081	0.112	0.119	0.059	0.029	0.027
NN-2	0.148	0.048	0.162	0.079	0.126	0.079	0.045	0.167	0.054	0.029	0.064
NN-3	0.168	0.042	0.175	0.127	0.103	0.054	0.059	0.130	0.075	0.047	0.022
NN-4	0.143	0.049	0.137	0.165	0.124	0.040	0.044	0.124	0.078	0.052	0.044
NN-5	0.174	0.029	0.225	0.134	0.058	0.066	0.077	0.132	0.062	0.025	0.018
NN-6	0.162	0.034	0.165	0.133	0.099	0.050	0.038	0.160	0.070	0.022	0.066
NN-7	0.179	0.056	0.117	0.153	0.091	0.039	0.022	0.170	0.042	0.082	0.046
NN-8	0.129	0.051	0.149	0.166	0.095	0.068	0.078	0.130	0.065	0.023	0.047
NN-9	0.149	0.041	0.134	0.132	0.090	0.067	0.093	0.085	0.080	0.091	0.038
NN-10	0.097	0.063	0.126	0.169	0.089	0.052	0.059	0.154	0.067	0.076	0.048
Average importance	0.147	0.046	0.156	0.138	0.096	0.059	0.062	0.137	0.065	0.047	0.042
Normalized importance	94.8%	30.6%	100.0%	89.5%	63.3%	38.6%	40.7%	88.4%	42.5%	31.4%	27.6%

PP = Perceived Performance; PFV = Perceived Functional Value; PV = Perceived Value; PS = Perceived Satisfaction; HAB = Habit; HM = Hedonic Motivation; CUR = Curiosity; SI = Social Influence; EA = Environmental Awareness; FC = Facilitating Condition; CV = Cost Value.

**Figure 4:**
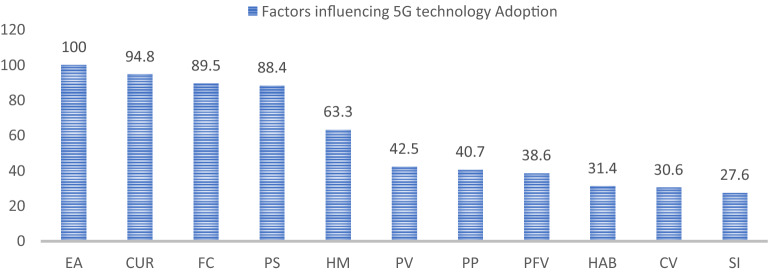
ANN Model.

## Discussion

**Figure 5:**
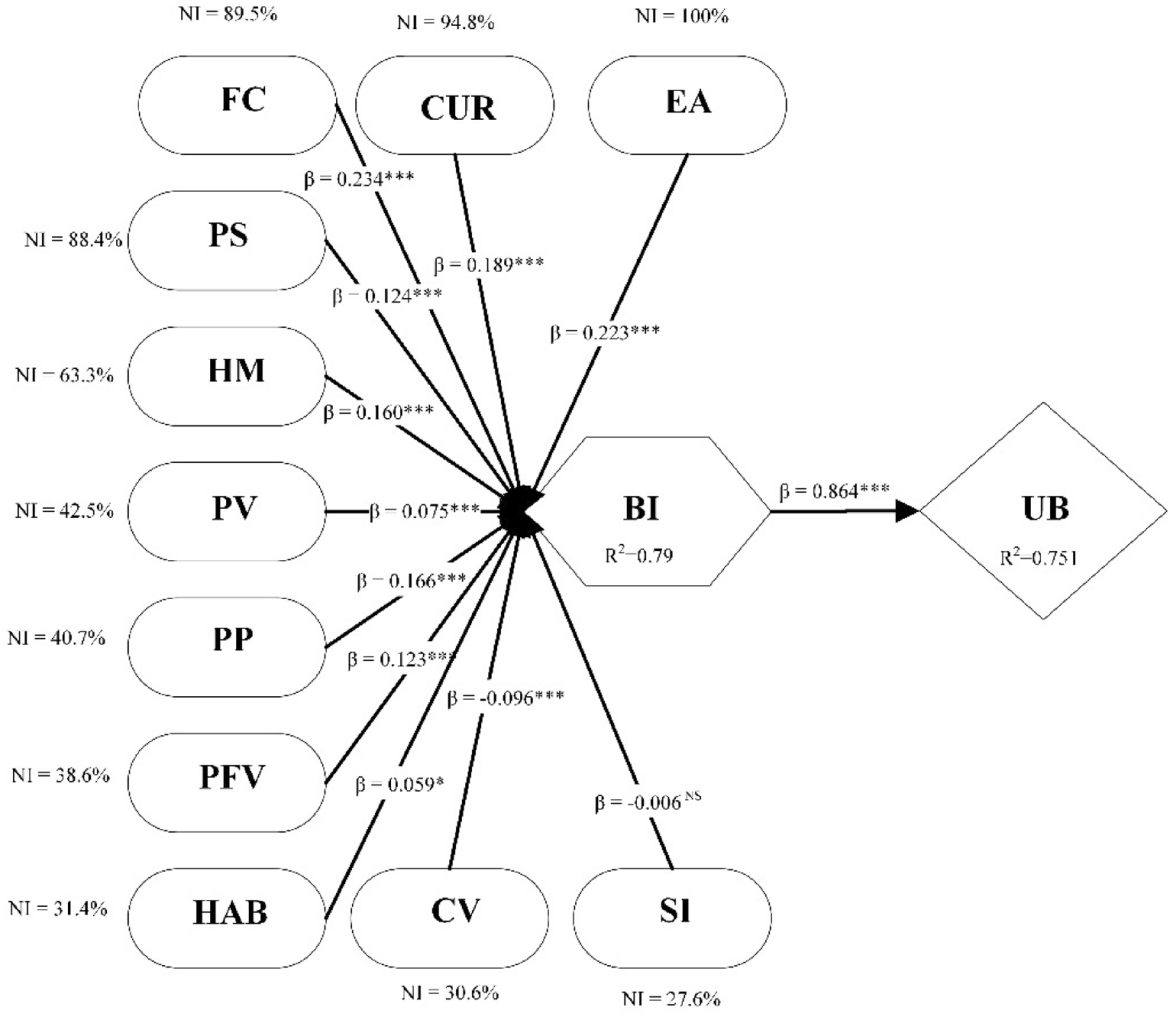
SEM-ANN results. Note: PP = Perceived Performance; PFV = Perceived Functional Value; PV = Perceived Value; PS = Perceived Satisfaction; HAB = Habit; HM = Hedonic Motivation; CUR = Curiosity; SI = Social Influence; EA = Environmental Awareness; FC = Facilitating Condition; CV = Cost Value. NI = normalized importance from ANN sensitivity analysis, β = Coefficients from SEM; R^2^ = Calculated in SEM.

This research reveals statistically significant 5G technology adoption factors within intrinsic, psychological, social, and economic dimensions. These factors can be the foundation for understanding consumer intention and behaviour in technology adoption. Environmental awareness, curiosity, facilitating conditions, and perceived satisfaction are the four most important factors behind 5G adoption (Fig. 5). It indicates that the rest of the factors can be improved and play a significant role if they evolve and improve carefully. Stakeholders need to focus accordingly to improve sales or the adoption rate of 5G.

This study reflects that all three intrinsic factors, i.e., perceived value, perceived functional value, and perceived performance, positively influence 5G adoption intention and are statistically significant. These findings are consistent with the previous studies, such as mHelth adoption 18, mobile internet adoption^23^, 5G purchase^10^, intention to purchase digital items^16^, and cellular technology adoption 2. Among these three intrinsic predictors, the PV with 42.5% normalized importance is the most significant predictor of this section. PV normalized importance is comparatively less than psychological, social, and economic predictors, which means 5G technology adopters are relatively less concerned with intrinsic factors.

This study revealed that psychological factors significantly influence 5G technology adoption intention. All psychological factors were found significant. Surprisingly, curiosity has never been used as a predictor in any technology adoption model, found to be the second most important predictor among all predictors with normalized importance of 94.8%. PS and HM ranked as the fourth and fifth most influential factors, with normalized importance of 88.4% and 63.3%, respectively. It implies that psychological factors are one of the most influential factors behind 5G technology adoption. Our findings are supported by existing literature mHealth adoption^18^, individual-level digital divide^28^, online healthcare^35^, mobile internet adoption 8, adoption of public Wi-Fi^37^, cellular technology adoption 2, and online game adoption^11^.

From the perspective of social factors, results revealed that social influence is not statistically significant and does not play any role in 5G adoption. These findings followed prior research^36^ intention and adoption of internet banking^20^ behavioural intention in using e-commerce in developing countries. The possible reason for insignificant SI is that mobile internet technology usage is solely a personal decision, and consumers discuss less about technological generations. Hence, they conceive less influence from fellow beings. On the other hand, shocking results for environmental awareness positively associated with BI emerged as the most important 5G adoption predictor with normalized importance of 100%. Clearly, consumers care about environmental factors; they are not only aware but highly sensitive regarding ecological change and their health. With these findings, we can conclude that people will likely stop using 5G technology if they find it hazardous to the environment and human health.

Further economic factors' influence has been checked on BI. Results revealed that facilitating conditions are statistically significant and positively associated with BI, consistent with previous studies mHealth adoption^18^, intention, and adoption of internet banking 40 but inconsistent with^28^ individual-level digital divide. According to the sensitivity analysis conducted with ANN, FC is the third most important factor among all predictors of 5G technology adoption. It means if consumers have 5G enable devices, they are likely to shift to the next generation of mobile internet technology. The contrary cost value is statistically significant but negatively associated with 5G adoption intention. These results were in accordance with previous research^59^. It means consumers are reluctant to adopt costly technologies.

Finally, we examined the influence of demographic factors included as control variables in our model, and gender has a significant negative effect on 5G use. In contrast, education and age have no discernible effect on how 5G technology is used. As a result, we may infer that age and education affect many technology adoption pieces of research. Nonetheless, these characteristics have little effect on the adoption of 5G technology. Consumers react similarly regardless of their age or academic level. The findings are the same as recent research^11^.

## Contribution and future recommendations

### Theoretical contribution

This is one of the first studies to examine customer attitudes toward 5G mobile internet technology adoption. Our work is a pioneer in adopting 5G technology by presenting an empirically validated model. Our study results provide several significant contributions to available knowledge on technology adoption, specifically 5G adoption. Firstly, we presented an integrated technology adoption model based on the UTAUT2. Our proposed technology adoption model has integrated four new variables: environmental awareness, curiosity, perceived functional value, and perceived value. It is an inestimable addition to existing literature.

Secondly, our suggested 5G adoption model based on UTAUT2 presents a better understanding of consumers' psyche. It is a useful addition to the prevailing literature to comprehend human behaviour regarding technology adoption. Our model can measure and identify the consumer intention to adopt newly invented digital technologies.

Thirdly, we have used a modern deep learning approach of SEM-ANN to test our model. ANN results are robust, and we can conclude more reliable and compact results with this approach. Sensitivity analysis was performed in the second stage using ANN based on the significant variables at the first stage (SEM) to rank the significant variables. The study revealed that R^2^ values in SEM analysis are 75.1%, while R^2^ results at the second stage (ANN) are 83.6%. Our two-stage deep learning approach explains the variance better and more comprehensively. If we compare our model and methodology results with contemporary technology adoption models, our model and technique outperform other models regarding explained variance^8,10^. Furthermore, if we compare our results with the baseline model UTAUT2, explained variance is higher than the baseline model. UTAUT2 explains 44% variance at BI and 35% at UI, along with moderated effects; it explained 55%, far less than our integrated model. Based on the results, we can say that as we have integrated new variables in the baseline version of UTAUT2, a new version of integrated UTAUT2 can explain human behaviour better than its previous version.

Fourthly, we added four new dimensions of human behaviour in the proposed technology adoption model. These four factors were significantly associated with 5G technology adoption, but two (EA and Curiosity) out of four, surprisingly, were the most influential factors behind 5G technology adoption in the sensitivity analysis. Suggest that these factors must be incorporated in TAM, especially when researchers study environment-related technologies.

Our proposed model is more comprehensive and concrete, covering all possible theoretical aspects of technology adoption, including psychological, social, intrinsic, and economic factors. These theoretical findings will serve as the foundation for future research on technology adoption.

### Practical implication

This study provides several empirical findings, which will be crucially helpful for the 5G technology provider telecommunication companies, advertisers, government officials, and key policymakers. Firstly, we found that cost value and 5G technology adoption negatively correlate and indicate that users are concerned about the cost of 5G technology. We suggest telecommunication companies take care of the cost factor whenever they launch their technology in a new country and retain their users in already launched sixty countries. They need to focus on technology quality and cost in parallel. Furthermore, service coverage is not the same in all areas, e.g., 5G services initially might not be available in remote areas. Companies need to declare that if a customer buys a 5G mobile internet package and does not enjoy the service because of the infrastructural development, customers will only charge for the services they enjoyed, i.e., 4G or 3G. It will stimulate the consumers' perceived value and functional value of services a telecom company provides, and they will be more satisfied and happily pay for it. Policymakers and government officials need to pay attention to the tax bracket when signing a contract with telecommunication companies. Whenever a new technology prevails in society, it opens several economic windows. Huge economic and revenue generation activities are associated with 5G technology^61^. Telecommunication companies and governments should analyze the ground reality before starting any economic activity based on 5G technology. According to our findings, fewer people shift to 5G technology if it is too expensive than 4G^59^. It is highly likely in developing countries because consumers are price-conscious, particularly those in developing countries^62^.

Secondly, our study results found that Environmental awareness, curiosity, facilitating conditions, and Perceived satisfaction are the most influential factors behind 5G technology adoption and usage behaviour. The marketing or advertising section of telecommunication companies may use these characteristics to their advantage while advertising and presenting their technology to new markets and increasing their consumer base in existing ones. They are encouraged to make advertisements that significantly stimulate the customer psyche in relation to the factors mentioned above. They also encourage the development of public awareness before entering a new market and stimulate consumer curiosity. Furthermore, factors with low-importance values need to focus on stakeholders, and consumers' intentions need to stimulate by improving these factors. Based on sensitivity analysis, these factors have a lot of grey areas to improve that can help achieve a maximum 5G sales or adoption rate.

Thirdly, our study findings will help policymakers make telecommunication policies, as environmental awareness is the most crucial factor in 5G technology adoption. Policymakers must remember that customers are worried about ecological factors regarding new technology adoption. People will likely stop using 5G technology if they find that it has a hazardous impact on the environment and human health. A public environmental awareness campaign can be launched favouring 5G technology to inform consumers that 5G technology is environmentally friendly. It does not have a considerable harmful impact on human health and will not affect the ecological system.

Fourthly, our study findings will guide telecommunication companies to shape their penetration strategy in new markets and capture significant shares. Management can consider different incentive packages to target different segments of the target market. For instance, companies may provide a free trial/use to consumers before purchasing a 5G connection or pay attention to the initial pricing. This study's results could help prepare for 5G technology implementation.

### Limitations and future recommendations

Although we tried to cover all necessary aspects of our topic in this study, our study still has limitations. First, we took our sample from one country. We recommend conducting the same study in different geographic boundaries to comprehend 5G adoption. Because the possibilities are that social, psychological, economic, or cultural factors fluctuate in other parts of the world. Second, income level can be a potentially influential factor behind consumer decision-making that can influence a consumer to adopt or reject 5G. So, future studies recommend capturing the consumer's income and checking its impact on the 5G adoption, which we have missed in our study. Third, we did not consider geographic factors' mediation or moderation role. Previous studies^8,24^ have also checked the moderation role of income, gender, age, and experience in technology adoption 8. Forth we did not investigate the mediation role of behavioural intention between independent variables and 5G usage behaviour. We also recommend exploring the mediation role of behavioural intention to understand users' behaviour. Last but not least, as sensitivity analysis revealed, environmental awareness is the most significant factor behind 5G adoption. Scholars are encouraged to conduct research on the ecological side of 5G and check the impact of 5G network signals on the ecological systems.

### Declarations

- We confirm that all methods were carried out in accordance with relevant guidelines and regulations. Humans who participated in this study are aware of the purpose of the study, and their confidential information has not to be shared with anyone.
- All study participants provided their written informed consent. Study data is used after the consent of participants. The questionnaire used in this study started with the declaration and purpose of the study.
- Experimental protocol was approved by the ethical review board of the Beijing university of technology.

## Supplementary Information

Supplementary Information.

## Data Availability

Data used in this study is available at the following link. https://docs.google.com/spreadsheets/d/1tEQazCBjuldf55A9iK3t-B3BlOWAepDI/edit?usp=sharing&ouid=104056982942615139504&rtpof=true&sd=true.
