# Supplementary material for: An integrated model of UTAUT2 to understand consumers' 5G technology acceptance using SEM-ANN approach
Source: Sci Rep. 2022 Nov 21;12:20056. doi: 10.1038/s41598-022-24532-8 (PMC9681779; doi:10.1038/s41598-022-24532-8)
Supplement: Supplementary file 1 — Supplementary Information. [file 41598_2022_24532_MOESM1_ESM.docx]

**An integrated model of UTAUT2 to understand consumers' 5G technology acceptance using SEM-ANN approach.**

**Sohaib Mustafa^1^, Wen Zhang^1^, Shahzad Anwar^2*^, Khalid Jamil^3^, Sehrish Rana^4^**

**^1^ College of Economics and Management, Beijing University of Technology, Beijing 100124, China.**

**^2*^MBA Department Kardan University Kabul, Afghanistan.**

**^3^ School of Economics and Management North China Electric Power University Beijing 102206, China**

**^4^ Government Islamia Graduate College for Women, Faisalabad, Pakistan.**

**sohaibmustafa44@gmail.com (Sohaib), zhangwan@bjut.edu.cn (W Zhang), s.anwar@kardan.edu.af (Shahzad)*, khalidjamil29@yahoo.com (Khalid), sehrishrana26@gmail.com (Sehrish)**

- 1. **Research context**

Considering our study's goal, we chose China to test our model. This choice is based on China being a pioneer in 5G technology. In the first half of 2019, China's Ministry of Industry and Information Technology noted that commercial 5G deployment grew steadily. China has granted four major telecom companies licenses for 5G network deployment. Over 400 million Chinese consumers will use 5G services by 2025, making China the world's largest 5G market. By November 2019, over ten available 5G-compatible smartphone models in China and 5G service is available for the whole country ^1^. The 5G network in China is the largest in the world. By 2020, China's 5G network covered over 600,000 base stations, bringing the total to more than 718,000 ^2^.

**Table 1. Measurement construct**

| **Variables** | **Measurement Items** | | **References** |
| --- | --- | --- | --- |
| Perceived performance | PP1 | I find 5G Internet useful in my daily life. | ^3,4^ |
|  | PP2 | Using 5G Internet helps me accomplish things more quickly |  |
|  | PP3 | Using 5G Internet increases my productivity. |  |
| Perceived Satisfaction | PS1 | I am satisfied with the speed of 5G internet. | ^5^ |
|  | PS2 | I am satisfied with the 5G performance. |  |
|  | PS3 | I am fully satisfied with 5G services. |  |
|  | PS4 | I feel satisfied by my experience of using 5G internet. |  |
| Perceived Value | PV1 | The use of the 5G Internet offers value for money. | ^6,7^ |
|  | PV2 | The use of the 5G Internet is beneficial to me. |  |
|  | PV3 | Despite my familiarity with 5G, the use of 5G Internet is worthwhile to me. |  |
|  | PV4 | Overall, the use of 5G Internet delivers me good value. |  |
| Perceived Functional Value | PFV1 | 5G has an acceptable standard of quality | ^8,9^ |
|  | PFV2 | 5G possesses a degree of quality that is satisfactory. |  |
| Social Influence | SI1 | Society members who are influential to me think that I must use 5G Internet | ^3,4^ |
|  | SI2 | Society members who influence my behaviour think I must use 5G Internet. |  |
|  | SI3 | Society members whose opinions I value prefer that I use 5G Internet. |  |
|  | SI4 | I am inspired by society members who use the 5G internet. |  |
| Habit | HT1 | It has become my habit to use 5G internet | ^3,4^ |
|  | HT2 | I am addicted to using 5G Internet. |  |
|  | HT3 | I must use 5G Internet. |  |
|  | HT4 | Using 5G Internet has become natural to me. |  |
| Facilitating Condition | FC1 | I have the resources necessary to use 5G Internet. | ^3,4^ |
|  | FC2 | I have the knowledge necessary to use 5G Internet. |  |
|  | FC3 | I will use 5G internet if I have a compatible device. |  |
|  | FC4 | 5G Internet is compatible with the devices I use. |  |
|  | FC5 | I can get help from a service provider when I have difficulties using 5G Internet. |  |
| Hedonic Motivation | HM1 | The use of the 5G Internet is fun. | ^3,4^ |
|  | HM2 | The use of the 5G Internet is enjoyable. |  |
|  | HM3 | The use of the 5G Internet is very entertaining. |  |
| Cost Value | CV1 | 5G Internet price is reasonable. | ^3,4^ |
|  | CV2 | The price I pay for 5G Internet is well-matched of its value. |  |
|  | CV3 | At the present cost, 5G Internet delivers a good value. |  |
| Curiosity | CT1 | I am curious to use 5G technology. | ^10,11^ |
|  | CT2 | I want to know more about 5G technology. |  |
|  | CT3 | I want to know if 5G works better than 4G technology. |  |
|  | CT4 | I want to know how much 5G is different from 4G technology. |  |
| Environmental Awareness | EA1 | Before adopting a technology, I consider its environmental effects. | ^7^ |
|  | EA2 | I prefer to use technology that is more environmentally friendly. |  |
|  | EA3 | I am concerned about destroying and wasting the earth's resources. |  |
|  | EA4 | I am aware of 5G radiations and their impact on the environment. |  |
| Behavioural intention | BI1 | I intend to continue using 5G Internet in the future. | ^3,4^ |
|  | BI2 | I plan to continue to use 5G Internet frequently. |  |
|  | BI3 | I will always try to use 5G Internet in my daily life |  |
| Usage Intention | UI1 | I will use 5G in the future. | ^3,4^ |
|  | UI2 | I will recommend 5G services to others. |  |
|  | UI3 | 5G internet services increase my willingness to use them. |  |

**Table 2. Linearity of relationships.**

|  | | ***Sum of Squares*** | ***df*** | ***Mean Square*** | ***F*** | ***Sig.*** |
| --- | --- | --- | --- | --- | --- | --- |
| BI * PP | (Combined) | 651.730 | 125 | 5.214 | 19.762 | 0.000 |
|  | Linearity | 496.645 | 1 | 496.645 | 1882.426 | 0.000 |
|  | Deviation from Linearity | 155.086 | 124 | 1.251 | 4.740 | 0.000 |
| BI * PFV | (Combined) | 388.327 | 35 | 11.095 | 19.745 | 0.000 |
|  | Linearity | 330.394 | 1 | 330.394 | 587.980 | 0.000 |
|  | Deviation from Linearity | 57.933 | 34 | 1.704 | 3.032 | 0.000 |
| BI * PV | (Combined) | 389.520 | 141 | 2.763 | 4.279 | 0.000 |
|  | Linearity | 53.261 | 1 | 53.261 | 82.506 | 0.000 |
|  | Deviation from Linearity | 336.259 | 140 | 2.402 | 3.721 | 0.000 |
| BI * PS | (Combined) | 761.902 | 187 | 4.074 | 33.968 | 0.000 |
|  | Linearity | 435.772 | 1 | 435.772 | 3633.098 | 0.000 |
|  | Deviation from Linearity | 326.131 | 186 | 1.753 | 14.618 | 0.000 |
| BI * HAB | (Combined) | 611.432 | 127 | 4.814 | 14.990 | 0.000 |
|  | Linearity | 290.000 | 1 | 290.000 | 902.943 | 0.000 |
|  | Deviation from Linearity | 321.433 | 126 | 2.551 | 7.943 | 0.000 |
| BI * HM | (Combined) | 554.353 | 76 | 7.294 | 19.476 | 0.000 |
|  | Linearity | 395.762 | 1 | 395.762 | 1056.738 | 0.000 |
|  | Deviation from Linearity | 158.590 | 75 | 2.115 | 5.646 | 0.000 |
| BI * CUR | (Combined) | 579.181 | 125 | 4.633 | 12.679 | 0.000 |
|  | Linearity | 335.204 | 1 | 335.204 | 917.257 | 0.000 |
|  | Deviation from Linearity | 243.977 | 124 | 1.968 | 5.384 | 0.000 |
| BI * SI | (Combined) | 610.948 | 167 | 3.658 | 10.728 | 0.000 |
|  | Linearity | 269.573 | 1 | 269.573 | 790.517 | 0.000 |
|  | Deviation from Linearity | 341.375 | 166 | 2.056 | 6.031 | 0.000 |
| BI * EA | (Combined) | 807.734 | 260 | 3.107 | 55.564 | 0.000 |
|  | Linearity | 408.351 | 1 | 408.351 | 7303.511 | 0.000 |
|  | Deviation from Linearity | 399.383 | 259 | 1.542 | 27.580 | 0.000 |
| BI * FC | (Combined) | 772.308 | 179 | 4.315 | 42.001 | 0.000 |
|  | Linearity | 562.869 | 1 | 562.869 | 5479.408 | 0.000 |
|  | Deviation from Linearity | 209.439 | 178 | 1.177 | 11.454 | 0.000 |
| BI * CV | (Combined) | 480.236 | 92 | 5.220 | 10.835 | 0.000 |
|  | Linearity | 268.019 | 1 | 268.019 | 556.339 | 0.000 |
|  | Deviation from Linearity | 212.218 | 91 | 2.332 | 4.841 | 0.000 |
| UB * BI | (Combined) | 784.683 | 91 | 8.623 | 116.886 | 0.000 |
|  | Linearity | 658.337 | 1 | 658.337 | 8923.963 | 0.000 |
|  | Deviation from Linearity | 126.346 | 90 | 1.404 | 19.030 | 0.000 |
| **Note**: PP= Perceived Performance; PFV=Perceived Functional Value; PV=Perceived Value; PS=Perceived Satisfaction; HAB=Habit; Hedonic Motivation; CUR=Curiosity; SI=Social Influence; EA=Environmental Awareness; FC=Facilitating Condition; CV=Cost Value; BI=Behavioral Intention; UB=Usage Behavior | | | | | | |

**Table 3. Cross loadings**

|  | **BI** | **CUR** | **CV** | **EA** | **FC** | **HAB** | **HM** | **PFV** | **PP** | **PS** | **PV** | **SI** | **UB** |
| --- | --- | --- | --- | --- | --- | --- | --- | --- | --- | --- | --- | --- | --- |
| **BI1** | 0.893 | 0.573 | 0.541 | 0.672 | 0.763 | 0.522 | 0.635 | 0.532 | 0.688 | 0.635 | 0.259 | 0.526 | 0.623 |
| **BI2** | 0.91 | 0.57 | 0.482 | 0.642 | 0.727 | 0.522 | 0.602 | 0.539 | 0.698 | 0.675 | 0.171 | 0.482 | 0.645 |
| **BI3** | 0.9 | 0.563 | 0.498 | 0.563 | 0.718 | 0.545 | 0.616 | 0.629 | 0.693 | 0.637 | 0.247 | 0.52 | 0.504 |
| **CUR1** | 0.608 | 0.861 | 0.784 | 0.445 | 0.625 | 0.665 | 0.544 | 0.45 | 0.543 | 0.509 | 0.191 | 0.632 | 0.583 |
| **CUR2** | 0.603 | 0.915 | 0.736 | 0.453 | 0.594 | 0.668 | 0.485 | 0.471 | 0.52 | 0.455 | 0.182 | 0.644 | 0.581 |
| **CUR3** | 0.503 | 0.884 | 0.658 | 0.384 | 0.521 | 0.649 | 0.421 | 0.365 | 0.456 | 0.388 | 0.102 | 0.622 | 0.509 |
| **CUR4** | 0.494 | 0.867 | 0.621 | 0.377 | 0.522 | 0.66 | 0.402 | 0.376 | 0.45 | 0.38 | 0.141 | 0.71 | 0.513 |
| **CV1** | 0.434 | 0.599 | 0.83 | 0.374 | 0.52 | 0.51 | 0.387 | 0.325 | 0.46 | 0.414 | 0.155 | 0.544 | 0.49 |
| **CV2** | 0.464 | 0.719 | 0.878 | 0.33 | 0.53 | 0.567 | 0.49 | 0.343 | 0.444 | 0.367 | 0.135 | 0.519 | 0.469 |
| **CV3** | 0.559 | 0.755 | 0.899 | 0.393 | 0.622 | 0.625 | 0.552 | 0.391 | 0.568 | 0.507 | 0.169 | 0.602 | 0.541 |
| **EA1** | 0.478 | 0.344 | 0.337 | 0.739 | 0.466 | 0.314 | 0.38 | 0.353 | 0.422 | 0.416 | 0.073 | 0.296 | 0.583 |
| **EA2** | 0.491 | 0.372 | 0.311 | 0.741 | 0.467 | 0.304 | 0.348 | 0.314 | 0.42 | 0.381 | -0.043 | 0.291 | 0.586 |
| **EA3** | 0.542 | 0.347 | 0.325 | 0.787 | 0.465 | 0.301 | 0.412 | 0.321 | 0.46 | 0.349 | -0.018 | 0.298 | 0.562 |
| **EA4** | 0.604 | 0.385 | 0.32 | 0.792 | 0.509 | 0.351 | 0.397 | 0.371 | 0.563 | 0.494 | -0.033 | 0.329 | 0.498 |
| **FC1** | 0.708 | 0.502 | 0.523 | 0.536 | 0.858 | 0.495 | 0.604 | 0.464 | 0.651 | 0.632 | 0.247 | 0.47 | 0.726 |
| **FC2** | 0.73 | 0.592 | 0.559 | 0.553 | 0.879 | 0.556 | 0.574 | 0.56 | 0.697 | 0.699 | 0.19 | 0.544 | 0.729 |
| **FC3** | 0.651 | 0.469 | 0.52 | 0.523 | 0.863 | 0.459 | 0.599 | 0.43 | 0.687 | 0.686 | 0.173 | 0.48 | 0.669 |
| **FC4** | 0.74 | 0.574 | 0.544 | 0.566 | 0.895 | 0.533 | 0.605 | 0.458 | 0.7 | 0.692 | 0.163 | 0.505 | 0.701 |
| **FC5** | 0.618 | 0.595 | 0.59 | 0.449 | 0.723 | 0.641 | 0.767 | 0.489 | 0.545 | 0.486 | 0.126 | 0.558 | 0.576 |
| **HAB1** | 0.483 | 0.659 | 0.624 | 0.352 | 0.526 | 0.894 | 0.543 | 0.356 | 0.445 | 0.422 | 0.119 | 0.717 | 0.503 |
| **HAB2** | 0.496 | 0.698 | 0.616 | 0.331 | 0.535 | 0.916 | 0.542 | 0.393 | 0.499 | 0.466 | 0.127 | 0.742 | 0.452 |
| **HAB3** | 0.471 | 0.678 | 0.552 | 0.328 | 0.514 | 0.89 | 0.483 | 0.422 | 0.477 | 0.459 | 0.143 | 0.701 | 0.455 |
| **HAB4** | 0.533 | 0.509 | 0.436 | 0.392 | 0.558 | 0.694 | 0.58 | 0.57 | 0.434 | 0.45 | 0.098 | 0.512 | 0.499 |
| **HM1** | 0.497 | 0.444 | 0.44 | 0.36 | 0.572 | 0.562 | 0.836 | 0.379 | 0.447 | 0.39 | 0.124 | 0.506 | 0.453 |
| **HM2** | 0.678 | 0.498 | 0.53 | 0.502 | 0.722 | 0.564 | 0.944 | 0.468 | 0.576 | 0.48 | 0.176 | 0.485 | 0.624 |
| **HM3** | 0.655 | 0.489 | 0.518 | 0.478 | 0.686 | 0.601 | 0.916 | 0.482 | 0.541 | 0.498 | 0.174 | 0.543 | 0.619 |
| **PFV1** | 0.552 | 0.452 | 0.376 | 0.383 | 0.494 | 0.483 | 0.424 | 0.922 | 0.483 | 0.466 | 0.255 | 0.404 | 0.497 |
| **PFV2** | 0.614 | 0.434 | 0.384 | 0.441 | 0.559 | 0.482 | 0.496 | 0.937 | 0.548 | 0.504 | 0.285 | 0.414 | 0.59 |
| **PP1** | 0.657 | 0.465 | 0.478 | 0.561 | 0.647 | 0.491 | 0.501 | 0.506 | 0.876 | 0.633 | 0.15 | 0.471 | 0.599 |
| **PP2** | 0.659 | 0.485 | 0.494 | 0.484 | 0.66 | 0.473 | 0.493 | 0.481 | 0.874 | 0.624 | 0.209 | 0.447 | 0.608 |
| **PP3** | 0.711 | 0.531 | 0.529 | 0.574 | 0.741 | 0.482 | 0.546 | 0.481 | 0.889 | 0.748 | 0.176 | 0.524 | 0.662 |
| **PS1** | 0.515 | 0.345 | 0.354 | 0.391 | 0.504 | 0.426 | 0.303 | 0.466 | 0.557 | 0.786 | 0.104 | 0.42 | 0.514 |
| **PS2** | 0.429 | 0.366 | 0.374 | 0.329 | 0.494 | 0.414 | 0.281 | 0.391 | 0.554 | 0.73 | 0.122 | 0.416 | 0.415 |
| **PS3** | 0.587 | 0.331 | 0.342 | 0.406 | 0.614 | 0.344 | 0.447 | 0.386 | 0.568 | 0.76 | 0.168 | 0.344 | 0.536 |
| **PS4** | 0.662 | 0.482 | 0.468 | 0.515 | 0.706 | 0.47 | 0.506 | 0.393 | 0.674 | 0.824 | 0.123 | 0.504 | 0.635 |
| **PV1** | 0.238 | 0.137 | 0.147 | -0.018 | 0.2 | 0.093 | 0.142 | 0.249 | 0.175 | 0.147 | 0.874 | 0.13 | 0.251 |
| **PV2** | 0.228 | 0.136 | 0.146 | 0.015 | 0.203 | 0.11 | 0.188 | 0.289 | 0.178 | 0.159 | 0.881 | 0.154 | 0.237 |
| **PV3** | 0.17 | 0.164 | 0.157 | -0.052 | 0.141 | 0.148 | 0.123 | 0.23 | 0.153 | 0.113 | 0.851 | 0.175 | 0.195 |
| **PV4** | 0.209 | 0.176 | 0.156 | 0.012 | 0.173 | 0.147 | 0.149 | 0.218 | 0.181 | 0.143 | 0.798 | 0.134 | 0.233 |
| **SI1** | 0.478 | 0.678 | 0.564 | 0.333 | 0.479 | 0.633 | 0.433 | 0.378 | 0.486 | 0.425 | 0.148 | 0.844 | 0.448 |
| **SI2** | 0.447 | 0.679 | 0.569 | 0.314 | 0.468 | 0.648 | 0.44 | 0.329 | 0.41 | 0.393 | 0.167 | 0.866 | 0.45 |
| **SI3** | 0.522 | 0.675 | 0.571 | 0.369 | 0.528 | 0.772 | 0.517 | 0.451 | 0.498 | 0.5 | 0.125 | 0.873 | 0.508 |
| **SI4** | 0.35 | 0.264 | 0.312 | 0.245 | 0.465 | 0.423 | 0.426 | 0.219 | 0.345 | 0.433 | 0.112 | 0.594 | 0.375 |
| **UB1** | 0.712 | 0.526 | 0.497 | 0.644 | 0.686 | 0.521 | 0.547 | 0.53 | 0.6 | 0.602 | 0.24 | 0.493 | 0.889 |
| **UB2** | 0.77 | 0.592 | 0.527 | 0.646 | 0.738 | 0.495 | 0.539 | 0.535 | 0.633 | 0.633 | 0.237 | 0.496 | 0.905 |
| **UB3** | 0.893 | 0.573 | 0.541 | 0.672 | 0.763 | 0.522 | 0.635 | 0.532 | 0.688 | 0.635 | 0.259 | 0.526 | 0.923 |


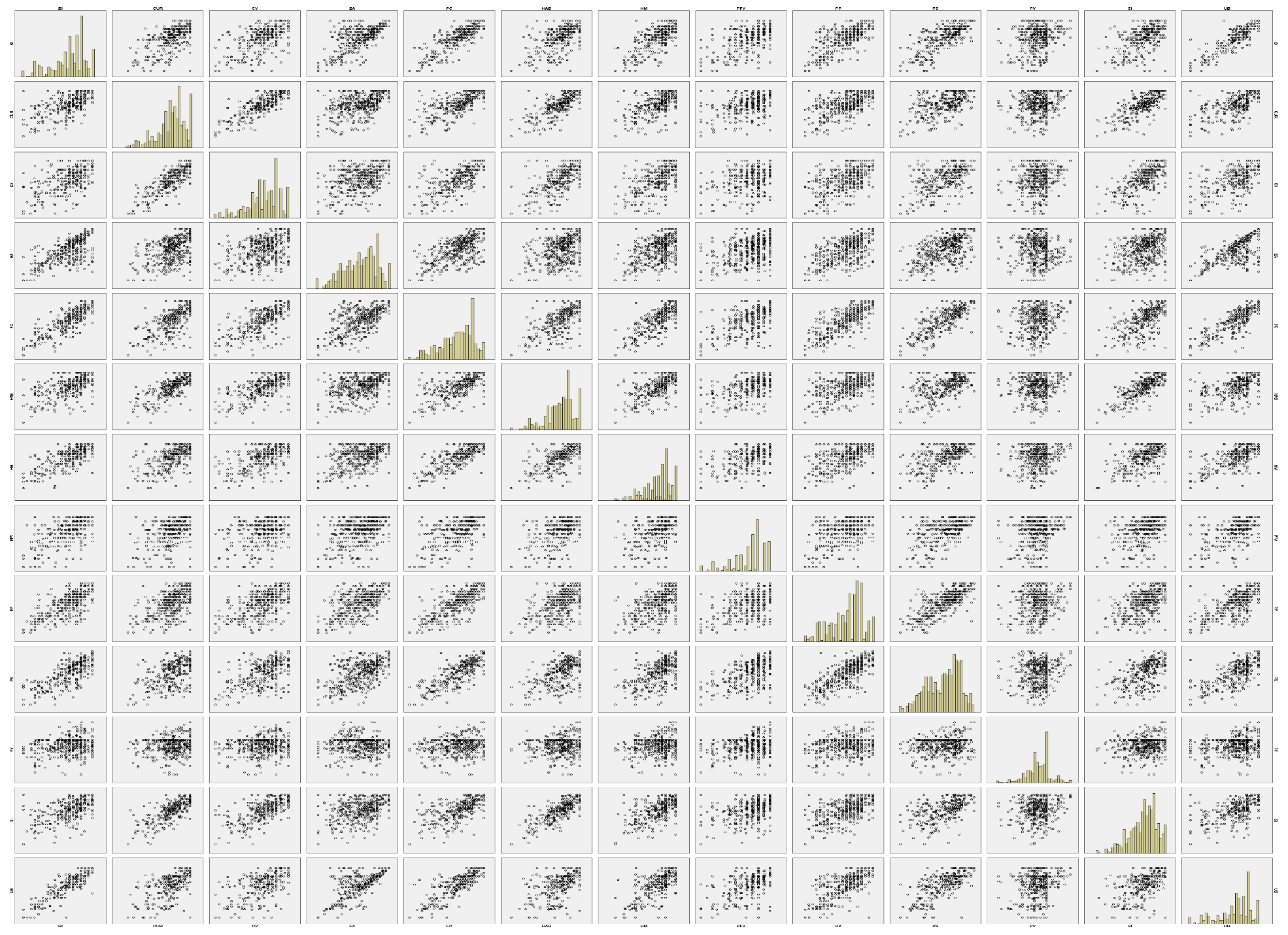


**Figure 1. Linearity assumption via scatter plot**

**References:**

1 The state council, T. p. s. r. o. C. *5G development in China*, <<http://english.www.gov.cn/news/videos/201908/01/content_WS5d4285f5c6d0c6695ff7e111.html>> (Aug-01-2019).

2 Meiping, G. *Tech Breakdown: What has China achieved in 5G development so far?*, <<https://news.cgtn.com/news/2021-04-14/Tech-Breakdown-What-has-China-achieved-in-5G-development-so-far--ZpeeToiJnG/index.html>> (2021).

3 Venkatesh, V., Thong, J. Y. L. & Xu, X. Consumer Acceptance and Use of Information Technology: Extending the Unified Theory of Acceptance and Use of Technology. *MIS Quarterly* **36**, 157-178, doi:10.2307/41410412 (2012).

4 Alam, M. M. D., Alam, M. Z., Rahman, S. A. & Taghizadeh, S. K. Factors influencing mHealth adoption and its impact on mental well-being during COVID-19 pandemic: A SEM-ANN approach. *J Biomed Inform* **116**, 103722, doi:10.1016/j.jbi.2021.103722 (2021).

5 Casaló, L. V., Flavián, C. & Guinalíu, M. Relationship quality, community promotion and brand loyalty in virtual communities: Evidence from free software communities. *International Journal of Information Management* **30**, 357-367, doi:<https://doi.org/10.1016/j.ijinfomgt.2010.01.004> (2010).

6 Kim, H.-W., Chan, H. C. & Gupta, S. Value-based Adoption of Mobile Internet: An empirical investigation. *Decision Support Systems* **43**, 111-126, doi:<https://doi.org/10.1016/j.dss.2005.05.009> (2007).

7 Shah, S. K., Zhongjun, T., Sattar, A. & XinHao, Z. Consumer's intention to purchase 5G: Do environmental awareness, environmental knowledge and health consciousness attitude matter? *Technology in Society* **65**, 101563, doi:<https://doi.org/10.1016/j.techsoc.2021.101563> (2021).

8 Yeh, C.-H., Wang, Y.-S. & Yieh, K. Predicting smartphone brand loyalty: Consumer value and consumer-brand identification perspectives. *International Journal of Information Management* **36**, 245-257, doi:10.1016/j.ijinfomgt.2015.11.013 (2016).

9 Kim, H.-W., Gupta, S. & Koh, J. Investigating the intention to purchase digital items in social networking communities: A customer value perspective. *Information & Management* **48**, 228-234, doi:<https://doi.org/10.1016/j.im.2011.05.004> (2011).

10 Marvin, C. B. & Shohamy, D. Curiosity and reward: Valence predicts choice and information prediction errors enhance learning. *J Exp Psychol Gen* **145**, 266-272, doi:10.1037/xge0000140 (2016).

11 Dahabiyeh, L., Najjar, M. S. & Agrawal, D. When ignorance is bliss: The role of curiosity in online games adoption. *Entertainment Computing* **37**, 100398, doi:<https://doi.org/10.1016/j.entcom.2020.100398> (2021).
